# A facile approach to prepare silicon-based Pt-Ag tubular dendritic nano-forests (tDNFs) for solar-light-enhanced methanol oxidation reaction

**DOI:** 10.1186/s11671-015-0791-9

**Published:** 2015-02-18

**Authors:** Chun-Ting Lin, Ming-Hua Shiao, Mao-Nan Chang, Nancy Chu, Yu-Wei Chen, Yu-Hsuan Peng, Bo-Huei Liao, Hung Ji Huang, Chien-Nan Hsiao, Fan-Gang Tseng

**Affiliations:** Instrument Technology Research Center, National Applied Research Laboratories, Hsinchu, 300 Taiwan; Department of Engineering and System Science, National Tsing Hua University, Hsinchu, 300 Taiwan; Department of Physics, National Chung Hsing University, Taichung, 402 Taiwan; Institute of Nanoscience, National Chung Hsing University, Taichung, 402 Taiwan; Division of Mechanics, Research Center for Applied Sciences, Academia Sinica, Taipei, 115 Taiwan

**Keywords:** Direct methanol fuel cell, Galvanic replacement reaction, Tubular dendritic nano-forests

## Abstract

In this paper, a facile two-step Galvanic replacement reaction (GRR) is proposed to prepare Pt-Ag tubular dendritic nano-forests (tDNFs) in ambient condition for enhancing methanol oxidation reaction (MOR) under solar illumination. In the first GRR, a homogeneous layer of silver dendritic nano-forests (DNFs) with 10 μm in thickness was grown on Si wafer in 5 min in silver nitride (AgNO_3_) and buffer oxide etchant (BOE) solution. In the second GRR, we utilized chloroplatinic acid (H_2_PtCl_6_) as the precursor for platinum (Pt) deposition to further transform the prepared Ag DNFs into Pt-Ag tDNFs. The catalytic performance and solar response of the Pt-Ag tDNFs toward methanol electro-oxidation are also studied by cyclic voltammetry (CV) and chronoamperometry (CA). The methanol oxidation current was boosted by 6.4% under solar illumination on the Pt-Ag tDNFs due to the induced localized surface plasmon resonance (LSPR) on the dendritic structure. Current results provide a cost-effective and facile approach to prepare solar-driven metallic electrodes potentially applicable to photo-electro-chemical fuel cells.

## Background

Direct methanol fuel cell (DMFC) has been deemed as one of the important power suppliers for renewable power applications due to the high energy-conversion efficiency thereof [1,2]. One of the major issues of DMFCs is the slow process of methanol oxidation reaction (MOR), which directly limits the efficiency of DMFC [3]. Traditionally, platinum (Pt)-based alloy has been used as common a catalyst in MOR. In the past two decades, many bimetallic catalysts have been proposed to enhance the efficiency of MOR, including Pt-Ru [4], Pt-Ag [5], Pt-Au [6], etc. Recently, metal-oxide-supported Pt catalysts, including Pt-TiO_2_ [7], Pt-ZnO [8], and PtRu-TiO_2_ [9], were proposed to boost methanol oxidation under ultraviolet (UV) illumination for photo-electrochemical fuel cells [9]. Although over 60% of enhancement on MOR has been realized under UV illumination (365 nm, 100 W) [8], seldom, reports discussed the solar enhancement toward MOR, especially on pure metallic catalysts. In this paper, a facile two-step Galvanic replacement reaction (GRR) is proposed to prepare Pt-Ag tubular dendritic nano-forests (tDNFs) in ambient condition for enhancing MOR under solar illumination.

In preparation of the aforementioned bimetallic catalysts, GRR was widely employed to provide a simple and cost-effective fabrication approach [10,11]. By utilizing the difference in the standard reduction potentials, replacement between two metals can be easily achieved at ambient condition. Many metal composites prepared by GRR have been reported, including Ag-Au [12,13], Pt-Au [14,15], Pd-Pt [16,17], Ag-Pt [18-21], Pd-Ag [22,23], Cu-Pd [24], and Cu-Ag [25]. However, most of the studies focused on the preparation of non-supported catalysts. The prepared catalysts suspended in the solution could be hardly collected and deposited on the electrodes in the electrochemical cells. Moreover, the effective electrochemical surface area of the non-supported catalysts could be greatly sacrificed due to the aggregation of nano-catalysts in brushing or printing process [4,26].

In order to prepare metallic nanostructures directly on supporting substrates, fluoride-assisted Galvanic replacement reaction (FAGRR) was proposed to synthesize three-dimensional metallic dendrites on silicon-based substrates [27-31]. Recently, Ye et al. reported a facile method for preparing self-assembled silver dendrites on silicon wafer in fluoride and silver nitride solution [27,29] for improving surface-enhanced Raman spectroscopy (SERS) [27-29,31]. However, the prepared silver dendrites could be easily contaminated by sulfur or oxygen to from Ag_2_O or Ag_2_S at ambient [32,33], which directly limits the applications for catalytic reactions.

In this paper, we propose the preparation of Si-based Pt-Ag tDNFs for solar-light-enhanced MOR by a two-step facile GRR at ambient without any energy input. This self-assembled Pt-Ag tDNFs not only benefit from the large aspect surface area provided by the Ag dendrites but also the localized surface plasmon resonance (LSPR) effect for the enhancement of methanol electrode-oxidation. Besides, the Pt outer shell of Pt-Ag tDNFs provides a protection to Ag and thus greatly enhances the stability of the prepared photo-electrodes.

## Methods

### Preparation of Ag DNFs and Pt-Ag tDNFs

**Figure 1:**
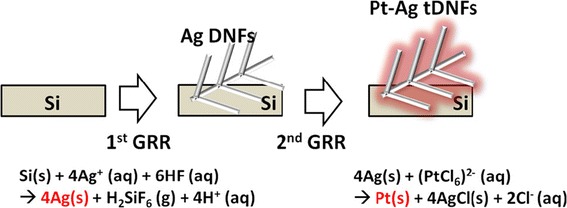
**The schematic drawing of the two-step GRR process.**

A two-step-GRR is proposed to prepare the Si-based Pt-Ag tDNFs as shown in Figure 1. In the first step of GRR, a fluoride-assisted GRR (FAGRR) [30] was adopted to prepare the Ag dendritic nanoforests (Ag DNFs) on planar silicon. In a typical process, n-type silicon (2 cm × 2 cm) was dipped into 2.5 mM silver nitride (AgNO_3_) + 25% (*v*/*v*) buffered oxide etchant (BOE) (comprising 34% NH_4_F and 7% HF) for 5 min at ambient. Following this, the sample underwent DI wash and N_2_ spray, and the Ag DNFs are ready as a scaffold for the next process. In the second step of GRR, the Si-based Ag DNFs were immersed into a 0.5 mM chloroplatinic acid (H_2_PtCl_6_) electrolyte for 5 min in ambient atmosphere for Pt deposition. The prepared samples were then immersed in 30% NH_4_OH for 1 h to remove AgCl precipitate [34]. After DI wash and dehydration bake at 105°C for another 5 min, the Pt-Ag tDNFs were ready for experiment. We also prepared a sample by sputtering 7 nm of Pt on one-step GRR-prepared Ag DNFs, which is denoted as S-Pt/Ag DNFs, for comparison.

### Surface morphology and material characterization

Scanning electron microscope (SEM; Hitachi FE 4300 in Instrument Technology Research Center (ITRC); Hitachi, Tokyo, Japan) and field emission transmission electron microscope (TEM; JOEL JEM2100F in National Chung Hsing University (NCHU); JOEL Ltd., Tokyo, Japan) were employed to investigate the surface profile of the prepared samples. Energy-dispersive X-ray spectroscopy (EDS) was used to analyze the elemental composition.

### Investigation on photo-enhanced electrochemical reactions

Electrochemical reactions were measured by a potentiostat (Autolab PGSTAT302N in ITRC) in a rectangular three-electrode reaction tank (500 mL) made by quartz. The prepared samples with projection area of 1 cm^2^ were used as the working electrodes, Pt-coated titanium mesh (25 cm^2^) as the counter electrode, and saturated calomel electrode (SCE) as the reference electrode. Cyclic voltammetry (CV) and chronoamperometry (CA) were used to evaluate the catalytic capability for methanol electro-oxidation. A solar simulator (SADHUDESIGN; class B; 400 to 1,000 nm; 1,000 W m^−2^) was employed for the illumination experiments. All chemicals used in this experiment were reagent grade. The resistance of DI water was 18.2 MΩ. All experiments were conducted at 22°C at ambient pressure.

## Results and discussion

### Surface morphology of Ag DNFs

**Figure 2:**
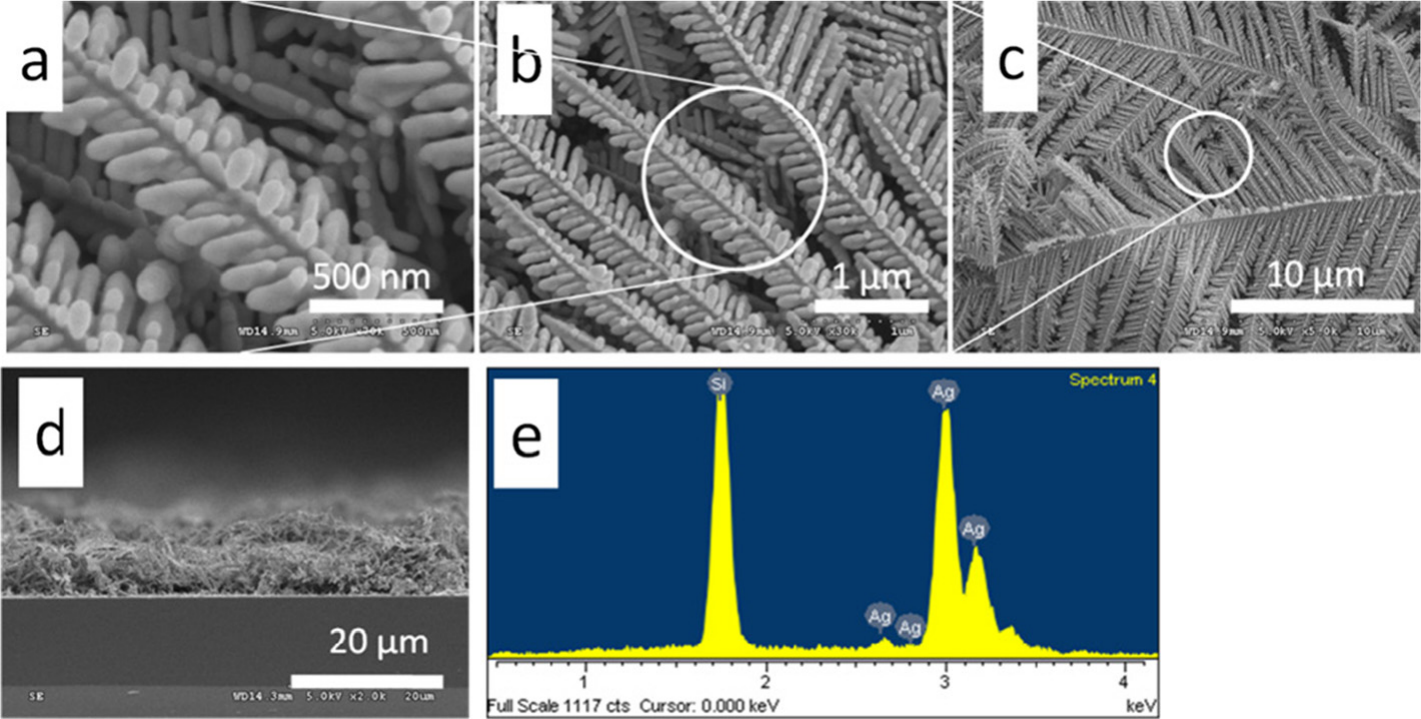
**The SEM images and EDS result of Ag DNFs. (a-d)** The SEM images of typical Ag DNFs grown on plane silicon in 2.5 mM AgNO_3_ and 25% (*v*/*v*) buffered oxide etchant (BOE) for 300 s. **(e)** The EDS result of the Ag DNFs shown in **(a)**.

**Figure 3:**
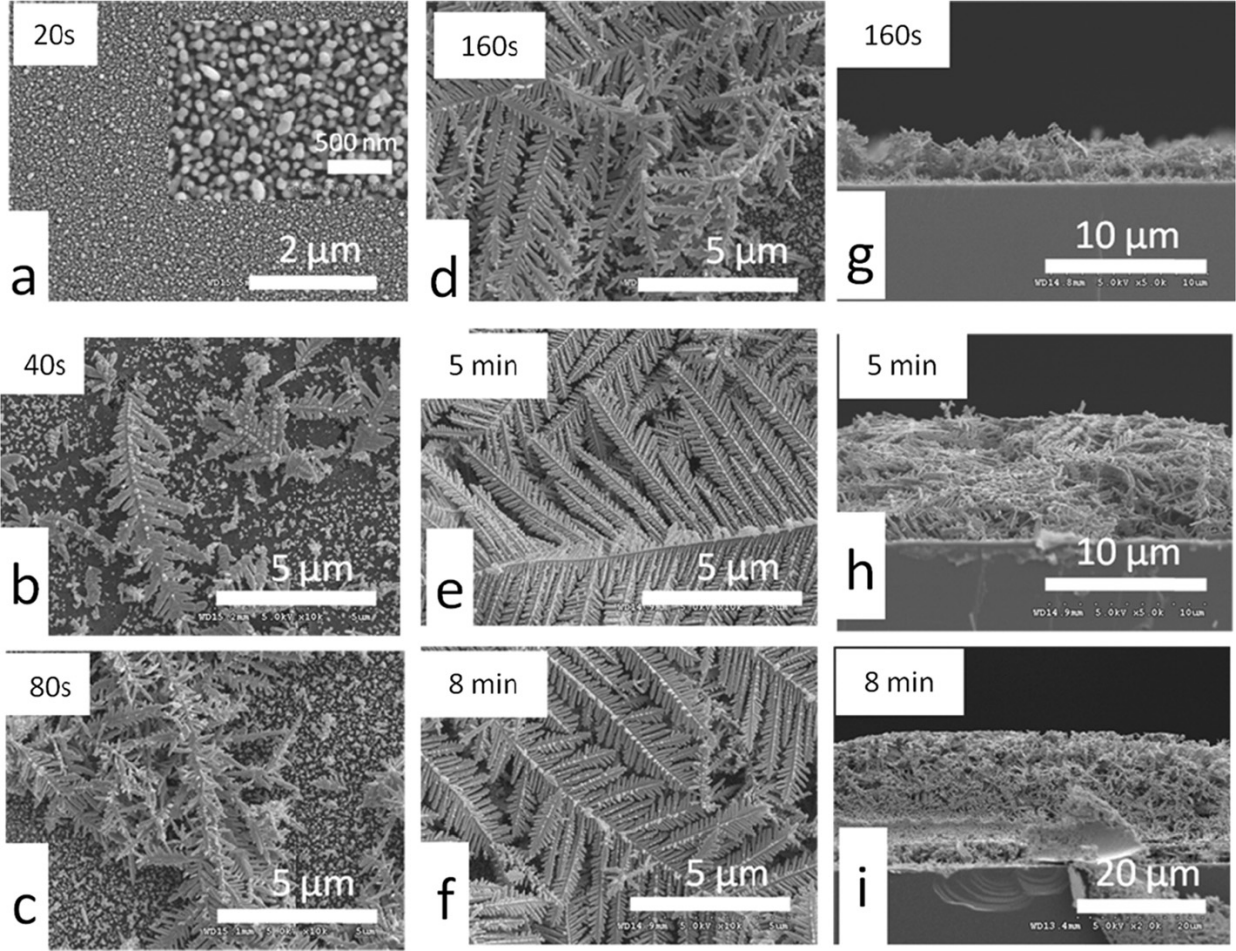
**The top (a-f) and side view (g-i) of SEM images of Si-based Ag nano-islands and Ag DNFs for various reaction durations.**

The SEM images shown from Figure 2a,b,c,d demonstrate the typical Ag DNFs on silicon substrate obtained by the first GRR. A 10-μm-thick Ag DNF layer was homogeneously formed on a plain silicon substrate within 5 min. It is found that oxygen signal was not detected in the as-prepared Ag DNFs in the EDS analysis (Figure 2e). This indicates our process is not favored for the formation of silver oxide. The growing process of Ag DNFs is schematically depicted in Figure 3. In the early stage of the reaction, Ag nano-islands dominated the surface of Si (Figure 3a). The dendritic structures started to appear after 40 s of the FAGRR and finally extended to the whole surface of Si (Figure 3b,c,d,e,f). The cross-section views (Figure 3g,h,i) demonstrate that the thickness of the Ag DNFs layer could reach 10 μm in 5 min and 20 μm in 8 min, respectively, which has not been reported in the previous studies on silicon-based silver dendrites [27-29]. The 10-μm-thick Ag DNFs (Figure 3i) were selected for the following experiments.

### Surface morphology of Pt-Ag tDNFs

**Figure 4:**
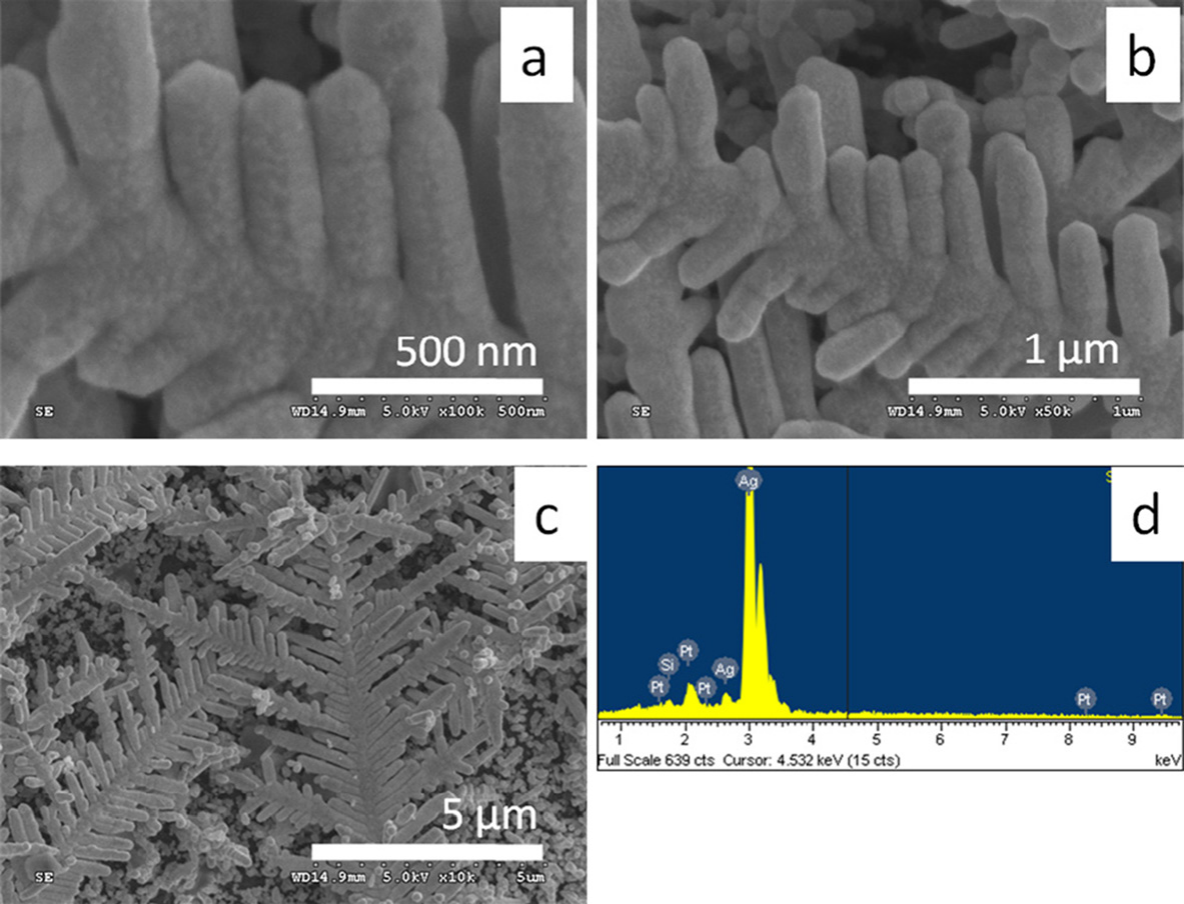
**The SEM images and EDS result of typical Pt-Ag tDNFs. (a-c)** The SEM images of typical Pt-Ag tDNFs. **(d)** The corresponding EDS result.

**Figure 5:**
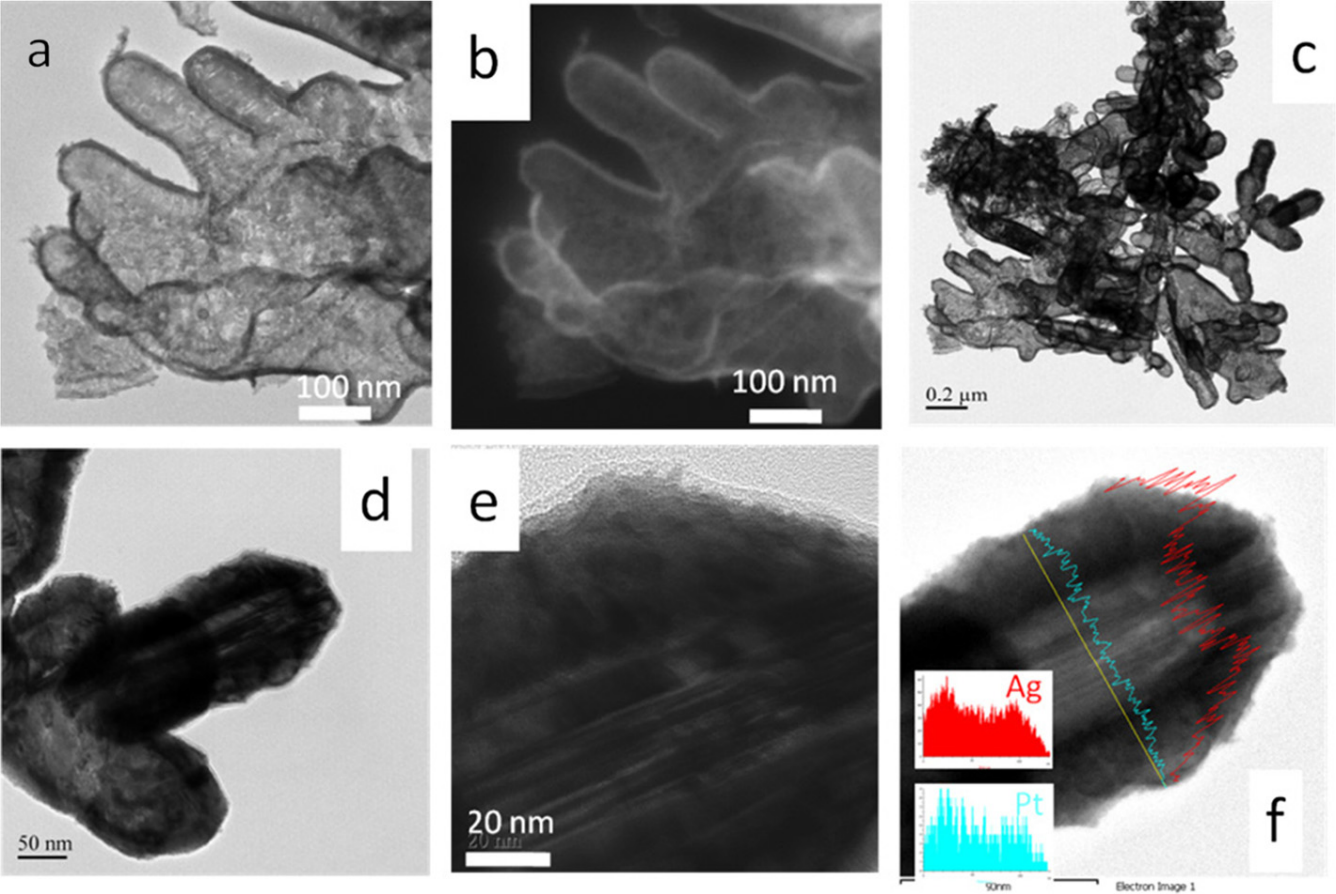
**STEM bright-field image, high-angle annular dark-field STEM image, TEM images, and EDS result. (a)** The STEM bright-field image of typical Pt-Ag tDNFs. **(b)** The high-angle annular dark-field (HAADF) STEM image of the same sample. **(c-e)** The TEM images in different magnifications of the same sample. **(f)** The corresponding EDS linear result.

Figure 4 shows the SEM investigations of typical Pt-Ag tDNFs and the corresponding EDS analysis result. The morphology of the Pt-Ag DNFs basically follows that of the Ag DNFs while the branches were broadened. The composition of Pt was verified by the EDS analysis (Figure 4d). Figure 5 shows the TEM and scanning transmission electron microscope (STEM) images of the Pt-Ag tDNFs. Dark nano-shells (approximately 20 nm in thickness) were found growing along with the fringe of Ag branches in the bright field images (Figure 5a,c,d,e). The nano-shells were found bright in the high-angle annular dark-field (HAADF) images in Figure 5b. Since HAADF images are highly sensitive to atomic-number contrast [35], we hence verified that a thin layer of Pt nano-shell was grown over the whole surface of Ag branches. The tubular nature of Pt-Ag tDNFs was verified by the EDS linear scan in Figure 5f. The main reason of the formation of the hollow structures could be attributed to the difference in diffusion rate between Ag in Pt and Pt in Ag during the GRR process, which is known as the Kirkendell effect [10]. Since the diffusion rate of Ag in Pt is faster than that of Pt in Ag, the voids in Ag domain accumulated to form pores beneath the outer shell and finally occupied the cores of the dendrites [10,21,36]. Besides, several parallel dark lines were observed growing along with the long axis of the composite dendrite in the Ag core region in Figure 5d,e. That provides an evidence to the Kirkendall growth of Pt, which is the main mechanism of the formation of multi-layered metallic nanostructures in GRR [36]. Similarly, hollow Pt-Ag hybrid structures prepared via GRR were also reported by other groups [19-21]. Zhang et al. used GRR to fabricate Pt/Ag hollow nanoboxes for methanol oxidation [19]. Bansal et al. replaced Ag nanocubes with Pt by GRR for H_2_ evolution reaction [20]. Also, Kim et al. prepared hollow Pt/Ag nanospheres by GRR for the degradation of rhodamine B [21].

### Photo-enhancement on methanol electro-oxidation

**Figure 6:**
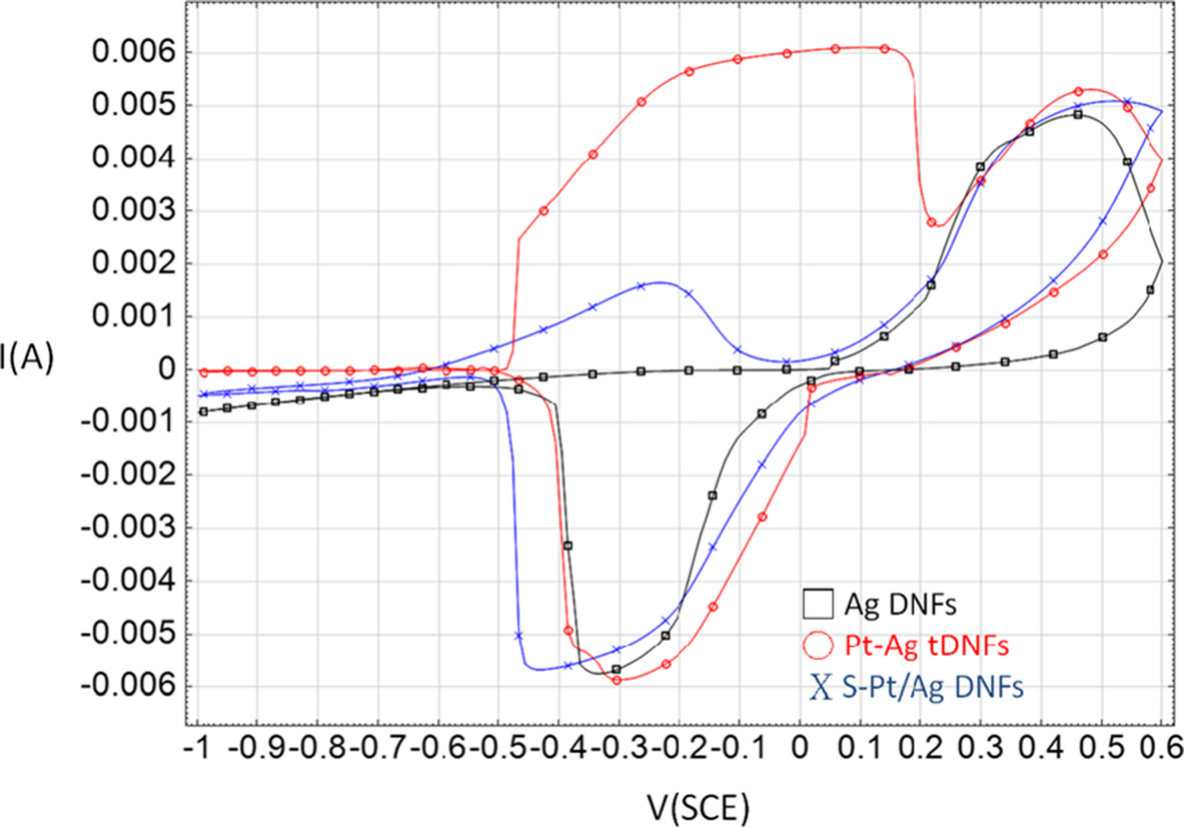
**Cyclic voltammograms of various working electrodes in 1 M CH**_**3**_ **OH + 0.5 M NaOH at 22°C in ambient condition.** Counter electrode: Pt-coated Ti mesh; reference electrode: saturated calomel electrode (SCE); scan rate = 30 mV/s.

**Figure 7:**
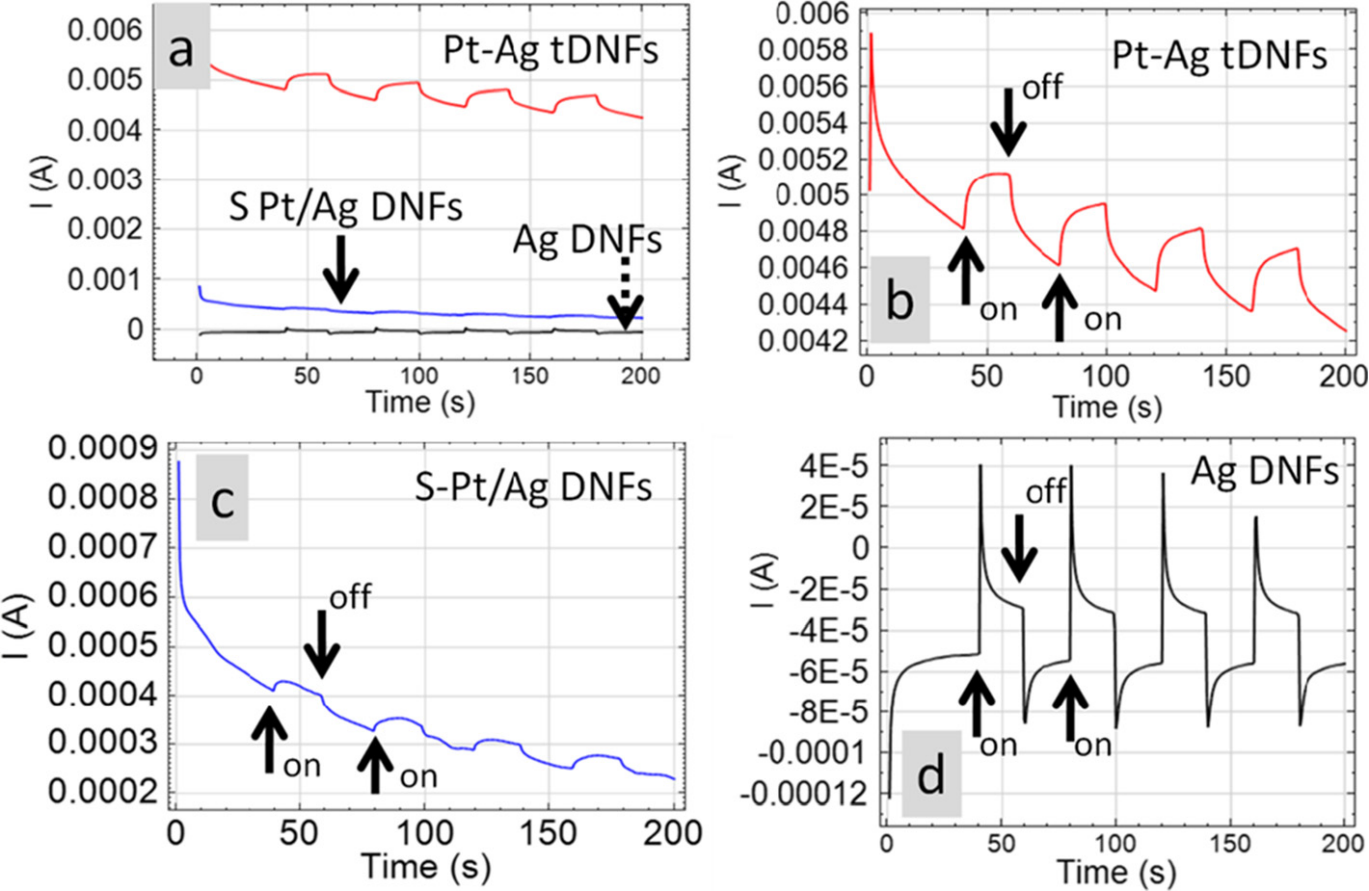
**The chronoamperometric (CA) on-off test of various working electrodes. (a)** The CA plots of various working electrodes. **(b-d)** The enlarged CA plots from **(a)** for Pt-Ag tDNFs, S-Pt/Ag DNFs, and Ag DNFs, respectively. The polarization potential is −0.3 V_SCE_. Counter electrode: Pt-coated Ti mesh; reference electrode: saturated calomel electrode (SCE). The illumination was conducted under a solar simulator (SADHUDESIGN; class B; 400 to 1,000 nm; 1,000 W m^−2^).

Figure 6 shows the cyclic voltammograms (CVs) of various working electrodes in 0.5 M NaOH + 1 M CH_3_OH. Both S-Pt/Ag DNFs and Pt-Ag tDNFs showed activity toward methanol electro-oxidation. The peak current densities of MOR of S-Pt/Ag DNFs and Pt-Ag DNFs are located at 1.65 and 6.10 mA cm^−2^, respectively. Also, it is found that Ag DNFs showed no activity toward methanol electro-oxidation. Only typical redox couple of Ag is observed in the CVs of the Ag DNFs, which coincides with the previous reports [5,37]. Figure 7 shows the on-off tests in CA with −0.3 V_SCE_ of bias applied to various working electrodes. All of the samples showed solar enhancement for MOR. In the first illumination cycle, the oxidation currents were boosted from 4.81 to 5.12 mA (6.4% of enhancement) and from 4.11 × 10^−4^ to 4.31 × 10^−4^ A (4.87% of enhancement) for Pt-Ag tDNFs and S-Pt/Ag DNFs, respectively (Figure 7b,c). Moreover, pulses of oxidation current were detected on Ag DNFs under illumination. In the first illumination cycle on the Ag DNFs, the oxidation current jumped from −5.14 × 10^−5^ to 4.10 × 10^−5^ A and was followed by a sharp decay to negative current region again within 2 s (Figure 7d). In the Ag DNFs, the strong electric field generated by LSPR results in the hot spots on specific confined areas of the dendritic structures. That helps deprive electrons from the adjacent methanol molecules and contribute to methanol electro-oxidation [38-40]. The sharp decay of oxidation current also suggests that only very limited methanol molecules in the hot spot region involved in the electron transfer process. In the cases of S-Pt/Ag DNFs and Pt-Ag tDNFs, the LSPR boosted the catalytic capability of Pt in the hot spot region and directly contributed to the oxidation current in the MOR. Also, more stable cyclic performance on photo-enhancement was found on Pt-Ag tDNFs when compared to that of S-Pt/Ag DNFs. That suggested the GRR provides better interface quality between Pt and Ag in Pt-Ag tDNFs.

**Figure 8:**
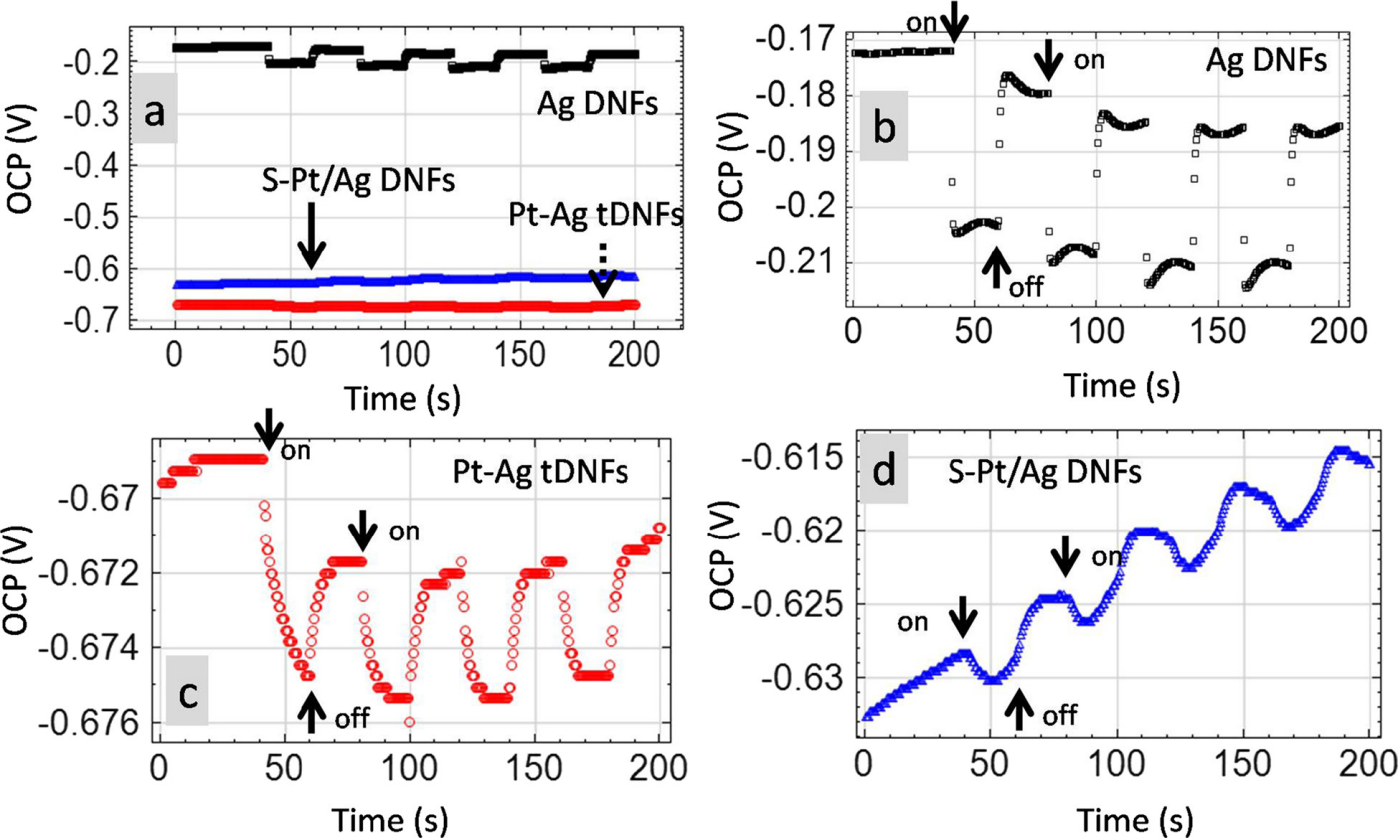
**The open**-**circuit potential (OCP) of various working electrodes during the on-off test under illumination. (a)** The OCP plots of various working electrodes. **(b-d)** The enlarged OCP plots from **(a)** for Ag DNFs, Pt-Ag tDNFs, and S-Pt/Ag DNFs, respectively. Counter electrode: Pt-coated Ti mesh; reference electrode: saturated calomel electrode (SCE). The illumination was conducted under a solar simulator (SADHUDESIGN; class B; 400 to 1,000 nm; 1,000 W m^−2^).

Figure 8 shows the open-circuit potential (OCP) variation under identical cyclic illumination performed in Figure 7. Negative shifts of OCP were found on all of the samples under illumination. The OCP variations at the first cycle of illumination were −0.06 V (−0.9%), −0.002 V (−0.3%), and −0.03 V (−19.2%) for Pt-Ag tDNFs, S-Pt/Ag DNFs and Ag DNFs, respectively. The trend of OCP variation coincides with that of the current enhancement. This negative shift in OCP could be explained as the change in the electronic state of the working electrodes, i.e., Pt-Ag DNFs, S-Pt/Ag DNFs, and Ag DNFs. Since Ag DNFs are well known as effective SERS substrates [27,41], we herein believe the strong LSPR effect played a crucial rule in the catalytic process [42]. Meanwhile, the Pt-Ag tDNFs show better cyclic stability than the S-Pt/Ag DNFs in OCP on-off measurement. That coincides with the results found in CA on-off test.

The mechanism of LSPR enhancement on electro-catalysis is a synergetic process, comprising plasmonic heating, magnification of local electromagnetic field, electron injection process, etc. [40-43]. Although more experiments are required to further elucidate the energy transfer process, current results provided a direct observation on the oxidation current enhancement and OCP variation under solar illumination in methanol electro-oxidation. The cost-effective and easily-prepared silicon-based Pt-Ag tDNFs are active to solar illumination and have high potential to serve as promising candidates for photo-electrochemical fuel cells.

## Conclusions

In this letter, focus is placed on the facile two-step GRR to prepare silicon-based Pt-Ag tDNFs in ambient condition for enhancing MOR under solar illumination. The FAGRR enables the fast growth of Ag NDFs on the silicon wafer within 5 min. Following that, the chloroplatinic acid further transformed the surface of Ag NDFs into Pt nano-shells and emptied the structure simultaneously within another 5 min. The prepared Pt-Ag tDNFs showed solar response (6.4% of enhancement on oxidation current) toward methanol oxidation. The solar response is attributed to the strong LSPR provided by the Ag DNFs. This cost-effective Pt-Ag tDNFs could be a promising candidate for photo-electrochemical fuel cells.
